# Chinese Baijiu: The Perfect Works of Microorganisms

**DOI:** 10.3389/fmicb.2022.919044

**Published:** 2022-06-16

**Authors:** Wenying Tu, Xiaonian Cao, Jie Cheng, Lijiao Li, Ting Zhang, Qian Wu, Peng Xiang, Caihong Shen, Qiang Li

**Affiliations:** ^1^Key Laboratory of Coarse Cereal Processing, Ministry of Agriculture and Rural Affairs, Sichuan Engineering and Technology Research Center of Coarse Cereal Industrialization, School of Food and Biological Engineering, Chengdu University, Chengdu, China; ^2^Luzhou Laojiao Co. Ltd., Luzhou, China; ^3^National Engineering Research Center of Solid-State Brewing, Luzhou, China; ^4^Postdoctoral Research Station of Luzhou Laojiao Company, Luzhou, China

**Keywords:** Chinese Baijiu, flavor substances, microorganisms, jiuqu, fermentation

## Abstract

Chinese Baijiu is one of the famous distilled liquor series with unique flavors in the world. Under the open environment, Chinese Baijiu was produced by two solid-state fermentation processes: jiuqu making and baijiu making. Chinese Baijiu can be divided into different types according to the production area, production process, starter type, and product flavor. Chinese Baijiu contains rich flavor components, such as esters and organic acids. The formation of these flavor substances is inseparable from the metabolism and interaction of different microorganisms, and thus, microorganisms play a leading role in the fermentation process of Chinese Baijiu. Bacteria, yeasts, and molds are the microorganisms involved in the brewing process of Chinese Baijiu, and they originate from various sources, such as the production environment, production workers, and jiuqu. This article reviews the typical flavor substances of different types of Chinese Baijiu, the types of microorganisms involved in the brewing process, and their functions. Methods that use microbial technology to enhance the flavor of baijiu, and for detecting flavor substances in baijiu were also introduced. This review systematically summarizes the role and application of Chinese Baijiu flavor components and microorganisms in baijiu brewing and provides data support for understanding Chinese Baijiu and further improving its quality.

## Introduction

Chinese Baijiu is an alcoholic beverage with a long history and is one of the great inventions of ancient China, which is also one of the six most popular distilled spirits in the world, including brandy, whiskey, vodka, rum, and gin (Xu et al., 2017b). Chinese Baijiu is usually brewed by solid-state fermentation with different grains as starter materials. Chinese Baijiu can be divided into different types according to different classification methods. Different types of baijiu contain many complex trace components. Jiuqu is a type of starter for baijiu fermentation, which is composed of raw materials, microflora, enzymes, and aromatic precursor substances. There are three types of microbial starter-jiuqu, including Daqu, Xiaoqu, and Fuqu. Their preparation and alcoholic fermentation are conducted under semi-controlled conditions. The flavor in Chinese Baijiu results from the presence of volatile and non-volatile substances, which are mainly produced by microbial metabolism during fermentation. Volatile substances include compounds such as esters, alcohols, acids, aldehydes, nitrogen-containing, and sulfur-containing compounds, and terpenes. These compounds exert important effects on the aromatic characteristics and quality of baijiu. Non-volatile substances are generally used as precursors of volatile substances, and their amount will affect the number of volatile substances in baijiu, thereby affecting its flavor and quality. Today, more than 2,400 chemicals have been identified in baijiu that contributes to flavor, some of which are beneficial to human health, such as short-chain fatty acids, peptides, and phenols (Sun et al., 2015; Fang et al., 2019; Xu et al., 2020; Jiang et al., 2021). Flavoring substances in Chinese Baijiu are detected using conventional detection technology, chromatographic technology, spectral technology, and other analysis and detection technologies, among which chromatographic technology is the most commonly used and effective (Jia et al., 2020a).

In the process of brewing Chinese Baijiu, fermentation is a complex microbial process. Many studies have shown that the raw materials and production process has a certain impact on baijiu flavor, and microorganisms from which play a key role (Zheng and Han, 2016; Jin et al., 2017; Wang et al., 2018b; Xu et al., 2017a). Microbial diversity is one of the important factors affecting baijiu flavor and microbial community in different jiuqu determines Chinese Baijiu flavor to a certain extent (Gou et al., 2015; Zheng and Han, 2016). Jiuqu provides microorganisms and enzymes to baijiu fermentation and significantly contributes to ethanol and flavor compound generation. Many studies revealed the structure and composition of microbial communities in jiuqu (Zheng et al., 2011). Furthermore, analysis indicates that jiuqu provides approximately 10 to 20% of the bacterial communities and 60 to 80% of the fungal communities to the baijiu fermentation (Wang et al., 2017c). The analysis of the Daqu microbial community showed that the formation of azines, esters, and aromatic compounds was affected by *Bacillus*, *Lactobacillus*, and *Aspergillus* (Jin et al., 2019). Jiuqu also provides abundant enzymes. Through the source-tracking analysis, Wang et al. (2020a) found that about 80% of carbohydrate hydrolases in baijiu fermentation were provided by jiuqu, and those enzymes were produced by *Aspergillus*, *Rhizomucor*, and *Rhizopus*. This can well indicate that the formation of baijiu flavor substances is inseparable from the interaction of jiuqu microbes. Recently, the role of microorganisms in regulating the formation of flavor compounds has been investigated. The contents of sulfur compounds, pyrazines, and acids in Chinese Baijiu were significantly increased when *Bacillus* was added to the fermentation microbiome. The initial fungal diversity also had a positive effect on fungal succession in fermentation, indicating the importance of the initial fungal diversity in promoting the formation of flavor compounds in Chinese Baijiu (Shen et al., 2020; Ji et al., 2022). Due to the open inoculation environment and complex fermentation environment, the quality of baijiu produced in different environments is different (Li et al., 2015). Many studies have found that microorganisms the from unique ecological environment are involved in the fermentation process of Chinese Baijiu, which promoted the formation of different flavor components in baijiu brewing (You et al., 2012; Calasso et al., 2016). Therefore, the baijiu fermentation process constructs the interaction among environment, microorganism, and baijiu flavor, in which microorganism is the key medium connecting environment and baijiu flavor (Wang, 2022). Many researchers have devoted themselves to examining the fermentation process, flavor components, microbial species and functions, and flavor detection methods for different flavors of baijiu. However, the main flavors of some baijiu and their aroma-generating mechanisms remain unclear, and there are relatively few comprehensive analyses of the characteristic flavors and microbial flora of the different flavors of baijiu. This review focuses on the introduction of different types of flavor components in Chinese Baijiu, and the types and functions of microorganisms involved in the process of brewing different flavors. In addition, methods of applying microbial technology to promote the synthesis of flavor substances and methods to detect flavor substances are also introduced. This paper provides a new and comprehensive perspective for the study of baijiu making, and provides data support for baijiu quality improvement.

## Flavor Types and Fermentation Microorganisms of Chinese Baijiu

Baijiu is an important part of the Chinese diet. Moderate drinking of baijiu plays an important role in people’s health and life quality. Previous studies found that regular consumption of mild to moderate baijiu could significantly enhance lipid metabolism and liver function (Zhi et al., 2022). Chinese Baijiu brewing uses different grains as fermentation raw materials. Jiuqu is used as a saccharification starter, and its main production steps are completed by solid-state fermentation, solid-state distillation, storage, and deployment. The main components of Chinese Baijiu are alcohol and water, accounting for 98% of the total weight, and there is usually less than 2% of other trace components, which included esters, aldehydes, ketones, acids, acetals, furans, terpenes, nitrogen compounds and sulfides (Hong et al., 2020). Esters are the main flavoring substances in Chinese Baijiu, accounting for more than 60% of the total flavor substances, of which ethyl acetate, ethyl butyrate, ethyl hexanoate, and ethyl lactate are the four main esters, accounting for approximately 75% of the total esters (Qian et al., 2019). Different types of baijiu have their unique microbiota and flavor due to their unique production techniques. Microbial activity plays a key role in baijiu brewing. Microorganisms not only directly determine the fermentation rate, but also convert nutrients into a variety of volatile flavor compounds. The microbial and flavor characteristics of various baijiu types are shown in Table 1.

**TABLE 1:** Microbial and flavor characteristics of various baijiu types.

Classification	Microbial characteristics	Flavor characteristics	References
Maotai-flavored baijiu	Filamentous fungi (*Aspergillus*), Yeast (*Pichia*), and Bacteria (*Bacillus*); Bacteria are most numerous; mold species are the most abundant.	Phenolic compounds: tetramethylpyrazine and syringic acid; high content of organic acids, aldehydes, ketones, alcohols, amino compounds, pyrazine, furans, and nitrogen compounds.	Xiong, 2005; Bokulich et al., 2012; Cai et al., 2019
Strong-flavored baijiu	Aroma-producing yeasts, bacteria, and molds; Bacteria mainly include *Bacillus*, *Streptomyces*, *Lysinobacter*, *Staphylococcus*, *Lactobacillus*, *Brevibacterium*, and *Brevibacterium*	mainly ethyl caproate, followed by ethyl acetate, ethyl lactate, ethyl butyrate and ethyl caproate	Wang et al., 2011; Bokulich et al., 2012
Light-flavored baijiu	Bacteria: *Bacillus* and *Lactobacillus*; Yeast: *Pichia thermophilus*, *Saccharomyces cerevisiae*, and *Isacchinia orientalis*	Aromatic compounds: ethyl acetate; esters, acids, alcohols, phenolic compounds; the main flavor substances are ethyl acetate, followed by ethyl lactate.	Sun et al., 2021; Tang et al., 2022
Rice-flavored baijiu	Bacteria: *Lactobacillus*, *Welchiella*, *Lactococcus*, *Acetobacter*, mainly *Lactobacillus*; Fungi: *Rhizopus*. Yeast: *Pichia anomala*, *Saccharomyces cerevisiae*, and *Issatchenkia orientalis*	Phenyl ethanol in equilibrium with ethyl acetate and ethyl lactate	Wu et al., 2019; Huang et al., 2020; Hu Y.L. et al., 2021
Miscellaneous-flavored baijiu	Bacteria: *Bacillus* and *Lactobacillus*; Fungi: *Paecilomyces*, *Saccharomyces*, and *Zygomycetes*	High concentrations of ethyl lactate, 2,3-butanediol, and ethyl hexanoate, followed by heptanoic acid, ethyl heptanoate, isoamyl acetate, octane, isobutyric acid, and butyric acid	Huang et al., 2021
Feng-flavored baijiu	*Naumovozyma*, *Rhizopus, Thermoascus*, *Aspergillus*, *Candida*, *Pseudeurotium*, *Saccharomycopsis*, *Pichia*, *Saccharomyces*, *Lactobacillus*, *Bacillus*, *Pediococcus*, and *Streptomyces*, among which *Naumovozyma* and *Lactobacillus* are dominant	Ethyl acetate, ethyl hexanoate, and isoamyl alcohol	Wang, 2008; Chen et al., 2020; Huang et al., 2021
Te-flavored baijiu	The dominant strains are *Saccharomyces*, *Pichia*, and *Galactomyces*	High concentrations of ethyl acetate and ethyl hexanoate, followed by ethyl propionate, ethyl valerate, ethyl heptanoate, and ethyl nominate	Li et al., 2017; Fu et al., 2021
Sesame-flavored baijiu	Mold, yeast, and bacteria	Sulfur compounds: dimethyl disulfide, trithiodimethyl, 3-(methylthio) propanal, furfural mercaptan, and furfural disulfide	Tamura et al., 2010, 2011
Laobaigan-flavored baijiu	Mold, yeast, and bacteria	Esters: ethyl acetate, ethyl lactate; low content of ethyl acetate, ethyl butyrate, palmitate, and linoleic acid	Fan Q. et al., 2019; Cui et al., 2020
Chi-flavored baijiu	*B. licheniformis*, *B. subtilis*, etc.	Acetic, lactic or butyric acid, etc.	Li et al., 2016; Cui et al., 2020
Fuyu-flavored baijiu	Bacteria: *Lactobacillus*, *Welbachia*, and *Bacillus*; Fungi: *Rhizopus*, *Candida*, *Pichia*, and *Aspergillus*.	Aromatic compounds: ethyl caproate, ethyl lactate and ethyl acetate	Kang et al., 2021

### Brief Introduction to Chinese Baijiu

Chinese Baijiu, also known as Laobaigan or Shaojiu, is a clear distilled baijiu obtained through a complex fermentation process. The literal translation of its name is ‘white alcohol,’ and it is an alcoholic beverage. As Chinese national baijiu, baijiu has a long history and unique brewing methods and occupies a very important position in the economy of China’s food industry. In 2020, Chinese Baijiu sales reached US$90.33 billion, with annual sales exceeding 10.7 billion liters. According to the ancient Chinese book ‘*Huangdi Neijing*,’ baijiu is the best medicine, and it emphasizes the importance of baijiu in medical care and disease treatment. In addition, the Compendium of Materia Medica noted that moderate consumption of alcohol can eliminate the feeling of cold, fatigue, and phlegm-dampness. This is by the results of some current clinical data analyses that indicated that moderate consumption of baijiu can accelerate blood circulation, improve the function of cardiovascular and circulatory systems, and decrease serum uric acid concentration and the risk of Alzheimer’s disease. Moderate alcohol consumption can also improve the health level of young people, and prevent cardiovascular disease by reducing blood lipid levels, platelet aggregation, and endothelial cell adhesion molecules. The researchers found that pyrazines, mainly tetramethylpyrazines, have antioxidant activity, boost immunity, and lower triglycerides (Yan et al., 2021). Other studies have found that baijiu intake has a potential cancer suppressor effect in animals. In mouse models, the spread and progression of breast cancer can be inhibited (Wang et al., 2022b). Ji et al. (2021) analyzed the effects of different baijiu on gut microbiota and host metabolism, and the results showed that volatile compounds have the potential to mitigate alcoholic liver disease by regulating gut microbiota and host metabolism. Therefore, moderate drinking is healthy and safe, but it is also important to limit high intake.

The production of Chinese Baijiu involves a complex fermentation system with multiple strains of microorganisms and many different types of raw grains, such as sorghum, rice, wheat, or millet, which are processed using unique fermentation technology. Chinese Baijiu is usually produced by natural solid-state fermentation that utilizes different microbial species (yeasts, bacteria, and molds), and saccharification and fermentation are conducted at the same time. Therefore, in such a production environment, a unique microbial community is used to create Chinese Baijiu, which results in its different flavor types and complex flavor characteristics (Jin et al., 2017; Liu and Sun, 2018). Hundreds of different types of baijiu in China are produced through different processes in different regions of China, as regional varieties. Chinese Baijiu can be classified according to the manufacturing technology, starter type, and product flavor. According to production technology, Chinese Baijiu can be divided into solid baijiu and semi-solid baijiu; according to the type of jiuqu, it can be divided into Daqu baijiu and Xiaoqu baijiu; and according to the flavor of the product, it can be divided into Maotai-flavored, light-flavored, Luzhou-flavored, rice-flavored, miscellaneous-flavored, te-flavored, feng-flavored, sesame-flavored, medicinal-flavored, chi-flavored, laobaigan, or fuyu-flavored baijiu. In recent years, there has been intense interest in studying the characteristic flavors of Chinese Baijiu, to excavate their main flavor and key flavor substances. The aroma and flavor of Chinese Baijiu are important factors that determine its type and quality. The formation of baijiu style characteristics is mainly related to approximately 2% of trace components in the baijiu body. Owing to the different flavors of Chinese Baijiu, its brewing process is complicated. It is produced mainly by solid-state microbial fermentation and brewing, solid-state saccharification fermentation, and solid-state retort distillation and brewing. It is also a key link shared by all flavored baijiu, and its unique brewing process distinguishes Chinese Baijiu from the other five major distilled spirits in the world.

Since ancient times, baijiu has been closely related to the life of the Chinese people. For the Chinese, baijiu is not only food but also a cultural heritage. At present, the characteristics of different types of Baijiu are still not clear. With the development of science and technology and the improvement of different brewing processes, baijiu with different flavors can be produced. However, Chinese Baijiu will require stricter and more accurate standards in the future to distinguish the myriad of flavor compounds in it.

### Flavor Types and Fermentation Microorganisms

**FIGURE 1:**
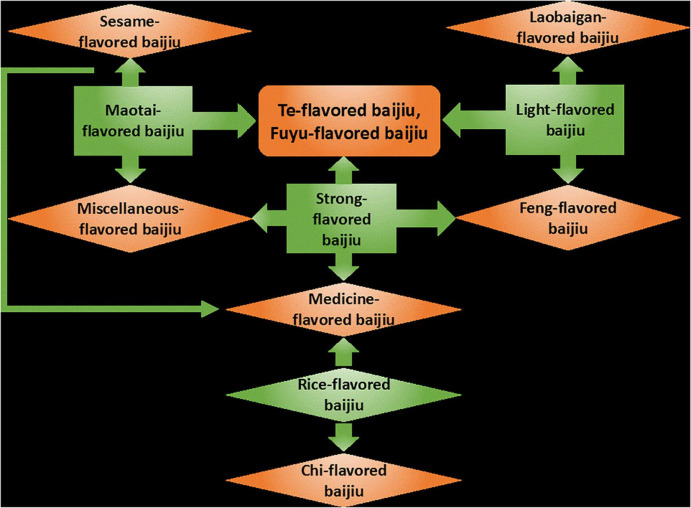
Relationships of 12 flavored baijiu types in China.

Chinese Baijiu can be divided into 12 types according to their flavors. Among them, Maotai flavor, Luzhou flavor, light flavor, and rice flavor are the basic four flavor types, and other baijiu is the extension of these four flavors (Zheng and Han, 2016; Xu et al., 2017b; Hu Y. et al., 2021). The relationship between the 12 flavored baijiu types is shown in Figure 1. The flavor characteristics of Maotai-flavored baijiu, Luzhou-flavored baijiu, and light-flavored baijiu are typical and representative, accounting for 60–70% of Chinese Baijiu. The production processes of the three baijiu are relatively standardized (Zheng and Han, 2016). Rice-flavor baijiu is produced from millet, which is inoculated Xiaoqu during fermentation, forming a unique flavor (Yin et al., 2020b). Chi-flavored baijiu was established based on rice-flavored baijiu and is different from rice-flavored baijiu due to different additives. Chi-flavored and rice-flavored baijiu are the only two types of baijiu produced by semi-solid fermentation. Sesame-flavored baijiu absorbed the flavor characteristics of three kinds of baijiu (Maotai flavor, Luzhou flavor, and light flavor) in the process of making jiuqu, and innovated the production of traditional baijiu by adding Fuqu (Jin et al., 2017). In addition, jiuqu brewed from barley and pea at low temperature was used in light-flavored baijiu, while laobaigan-flavored baijiu is made from pure wheat under medium temperature. A variety of jiuqu mixtures are usually used in the brewing of other baijiu flavors (Feng-flavor, Medicine-flavor, Te-flavor, etc.). For example, Xiaoqu made by adding some herbs is used to make baijiu with medicinal flavor.

#### Maotai-Flavored Baijiu

The four main steps in the production of Maotai-flavored baijiu are jiuqu making, alcoholic fermentation, solid-state distillation, and aging. In Maotai-flavored baijiu, sorghum (as a raw starch) and a unique Daqu (high-temperature Daqu) are mixed and fermented naturally in an open environment. Two-stage alcohol fermentation (including heap fermentation and cellar fermentation) occurs without directly or indirectly adding edible alcohol or non-self-fermented color, aroma, or taste substances (Jin et al., 2017; Liu and Miao, 2020). Maotai and Langjiu, are the most representative baijiu of Maotai-flavored baijiu, which provide a soy sauce-like aroma, full taste, and long-lasting aroma. To date, 528 compounds have been identified from Maotai-flavored baijiu (Zhu et al., 2007; Liang et al., 2019). Although over 30 major flavor substances have been identified in Maotai-flavored baijiu, the main aroma components remain inconclusive. Studies have shown that aromatic compounds (phenolic compounds) are its representative flavor, mainly tetramethylpyrazine and syringic acid, and a small number of amino acids, organic acids, and esters (Xiong, 2005). The contents of organic acids (acetic acid and lactic acid), aldehydes, ketones, alcohols, amino compounds, and nitrogen compounds in Maotai-flavor baijiu are high, while the total ester content is low. It is worth noting that the content of pyrazine and furan is also much higher than other baijiu (Wang et al., 2019).

Maotai-flavored baijiu has a unique fermentation process. It mainly uses high-temperature Daqu and combines the enrichment of various microorganisms in the natural fermentation environment to produce a variety of enzymes (mainly saccharifying enzymes) and microbial metabolites. Filamentous fungi, yeast, and bacteria work closely together in Maotai-flavored baijiu fermentation, and the enzymes produced by filamentous fungi degrade raw materials into fermentable sugars, which become substrates for yeast and bacteria to produce ethanol and other volatile compounds (Chen B. et al., 2014; Zheng and Han, 2016). When monitoring the microbial community in the solid fermentation substrate of Maotai-flavored baijiu, the researchers have found that the abundance of lactic acid bacteria was relatively high, so the quality and flavor of Maotai-flavored baijiu may be closely related to a variety of lactic acid bacteria (Wu et al., 2020a). In addition, reducing sugar is the key driving factor of microbial succession in heap fermentation, while acidity, alcohol, and temperature are the main driving factors of pit fermentation (Hao et al., 2021). Researchers also found that Daqu provided 95.6% of bacteria and 28.10% of fungi in Maotai-flavored baijiu fermentation, respectively. The environment (air, indoor floor, and tools) provided 71.9% of fungi (mainly *Pichia*) in Maotai-flavored baijiu fermentation, indicating that *Pichia* from the environment was also the dominant fungal genus involved in the fermentation process (Zhang et al., 2021). Environmental microbes play an important role in controlling and promoting the formation of microbial communities in baijiu fermentation. When high-temperature Daqu was used as a fermentation agent, the main microorganisms were bacteria, mold, and yeast, of which bacteria had the largest number, followed by mold and yeast. The most diverse species were fungi, followed by bacteria (Zuo et al., 2020). Wang et al. (2008) used phenotypic and conventional biochemical taxonomy to isolate and identify the microbial community structure of Maotai Daqu, and found that the bacterial genera included *Bacillus*, *Acetobacter*, *Lactobacillus*, and *Clostridium*, of which *Bacillus* has the largest number, *Bacillus* is the most important functional bacteria in the fermentation of Maotai-flavor baijiu (Zuo et al., 2020); the mold was present as mainly *Aspergillus*, and also with *Mucor*, *Rhizopus*, *Monascus*, and *Trichoderma*. Yeast included *Saccharomyces* spp., *Candida* spp., *Pichia* spp., and *Torula* spp. The most commonly isolated yeasts were from the genus *Saccharomyces*. The distribution of microbial species varies with the maturity stage of the Daqu. In high-temperature Maotai Daqu, the composition of microorganisms at different temperatures is different. At lower temperatures, yeasts, molds, and certain bacteria will accumulate in large quantities and will produce high levels of enzymes and other metabolites. Enzymes can hydrolyze proteins into various amino acids, and can also participate in the Maillard reaction and other chemical reactions with amino acids to produce the unique Maotai flavor. The microbiota plays an important role in the formation of flavor substances in the fermentation process. However, the flavor components and their proportion in Maotai-flavored baijiu are still uncertain. The mechanism of microbial diversity regulating the formation of flavor substances is complex. Thus on the research of Maotai-flavored baijiu is very challenging (Liu and Miao, 2020).

#### Strong-Flavored Baijiu

Strong-flavored baijiu is well known for its strong fragrance and sweetness, and it is the most popular type of Chinese Baijiu, also called Luzhou-flavored baijiu. The raw materials used in the fermentation of Luzhou-flavored baijiu are mixed with a variety of grains, mainly sorghum. Its production mostly adopts the process of steaming and slag, and fermentation occurs in old cellars, although there are also artificially cultivated cellars (Liu et al., 2017). Luzhou laojiao baijiu and Wuliangye baijiu are the most representative of the Strong-flavored baijiu, which possesses the characteristics of rich aroma, soft taste, and endless aftertaste. The most representative aromatic compounds in Luzhou-flavored baijiu are ethyl hexanoate, which is harmoniously balanced with ethyl lactate, ethyl acetate, and ethyl butyrate (Wang et al., 2020d). In addition, the acid content in Luzhou-flavored baijiu changed with the fermentation stage of base baijiu. The acetic acid, lactic acid, butyric acid, and hexanoic acid in the last mature Luzhou-flavored baijiu were stable, accounting for about 90% of the total acid content. Ethyl hexanoate is an ethyl ester that can produce an apple flavor and is one of the most important esters in Luzhou-flavored baijiu. The content of ethyl hexanoate, the main aromatic substance of strong-aroma baijiu, exceeds 200 mg/L in high-quality Luzhou-flavored baijiu.

Luzhou-flavored baijiu is usually produced by a typical natural solid fermentation method using Daqu as the main saccharifying agent. The fermentation process is generally anaerobic and conducted in pit mud. The composition of the flavor compounds in Luzhou-flavored baijiu, like other types of baijiu, is determined by the diversity of microorganisms. In the traditional fermentation process, ethyl hexanoate in Luzhou-flavored baijiu is mainly produced by aroma-producing yeasts, bacteria, and molds with high esterification ability in the mud pit in the late fermentation stage. In Luzhou-flavor Baijiu, it is important because bacteria can produce caproic acid and then regenerate it into ethyl caproic acid. Wang et al. (2020f) studied the fermentation pit mud and found that *Syntrophomonas*, *Methanobacterium*, and *Methanocorpusculurn* were conducive to the production of caproic acid. Although they are not directly involved, they provide possible environmental factors for caproic acid production. Zou et al. (2018) reviewed the species diversity and metabolism of *Clostridium* spp. Therefore, the presence of *Clostridium* can promote the solid-state fermentation process of baijiu (Bokulich et al., 2012). The research on microorganisms in Luzhou-flavored baijiu originally began in the 1960s. Today, many microbial strains have been identified and determined. As far as bacteria are concerned, there are roughly 34 bacterial genera, among which *Bacillus*, *Streptomyces*, *Lysinobacteria*, *Staphylococcus*, *Lactobacillus*, and *Brevibacterium* were the most important, and other new bacteria have also been detected (Wang et al., 2011). According to previous research (Endo and Okada, 2005; Chen et al., 2011), it is known that the microorganisms that can metabolize ethyl hexanoate esterase in nature mainly include bacteria, filamentous fungi, and yeast, many of which secrete extracellular esterase that catalyzes the synthesis of ethyl hexanoate. Some researchers have found through sensory-guided fractionation and component analysis that there are 43 volatile compounds (mainly esters) in Luzhou-flavored baijiu that seem to contribute to its sweetness, among which ethyl hexanoate and hexyl hexanoate have been identified. Esters and ethyl 3-methyl butyrate are the main contributors to the sweetness of baijiu (Tang, 2021). These studies not only broaden our understanding of baijiu sweetness but also highlight the main contribution of volatile compounds to the perception of baijiu sweetness. There is no doubt that the Ethyl caproate is one of the most important factors in determining the quality of Strong-flavored baijiu. In recent years, increasing the number of esters has been the main goal to improve the flavor of some aromatic baijiu. Zhao et al., Yin et al., and Yan et al. improved the characteristic flavor of Luzhou-flavored baijiu by enrichment of specific microorganisms and overexpression of related microbial metabolic genes (Zhao et al., 2017; Yan and Dong, 2018; Yin et al., 2019).

#### Light-Flavored Baijiu

The alcoholic fermentation of light-flavored baijiu, also known as light-flavored baijiu, is conducted in clay tanks, unlike Maotai-flavored baijiu and Luzhou-flavored baijiu, which are carried out in mud cellars. Light- flavored baijiu utilizes sorghum and wheat as the raw material and is brewed by steaming twice with slag cleaning technology. The production process of light-flavored baijiu involves a solid, open, natural fermentation, and therefore, the fermentation microorganisms of the light-flavored baijiu not only are derived from jiuqu but also derived from the surrounding environment. The most representative product of light-flavored baijiu is Fenjiu, and there is also Erguotou baijiu, with its mellow sweetness and fresh aftertaste. In many studies, it was determined that most flavor substances in light-flavored baijiu were synthesized at the late fermentation stage, and its metabolites were esters, alcohols, alkenes, sulfides, and ketones, furans, aldehydes, nitrogen compounds, alkanes and so on. The aromatic compound ethyl acetate is one of the main characteristic aromatic substances, which is balanced with a considerable amount of ethyl lactate. The proportion of n-propanol and isobutanol in alcohols was higher, which had a great influence on the flavor characteristics of baijiu (Liu and Miao, 2020). In addition, acetic acid and lactic acid are the main acids, with concentrations of 1 and 0.28 g/L, respectively, accounting for about 98% of the total acid content (Fan et al., 2018; Wei et al., 2020). Moreover, the ester acid ratio of light-flavored baijiu was higher than that of Luzhou-flavored baijiu (Yu et al., 2014).

The jiuqu brewed in light-flavored baijiu is produced using traditional fermentation techniques. The ingredients in the jiuqu (barley, peas, millet, and so on) and their production environment (tools, soil, air, and machinery) contain naturally occurring microorganisms. The functions of the primary microbial groups participating in the process of brewing light-flavored baijiu have been widely reported, and it has been said that the main bacterial genera in light-flavored baijiu are *Bacillus* and *Lactobacillus*. The role of *Lactobacillus* is to produce a large number of organic acids, while *Bacillus* can promote the production of unique flavor components. Fan G. et al. (2019) detected 72 volatile compounds, mainly alcohols, esters, aldehydes, alkenes, and alkanes, from Daqu by solid-phase microextraction coupled with gas chromatography-mass spectrometry. In addition, through high-throughput sequencing, it was found that the bacteria mainly came from *Pantoea*, but decreased with the increase of age. The bacteria of *Lactobacillus* and *Weissella* increased, while *Pichia* remained unchanged. The fermentation time of light-flavored baijiu is closely related to the change in the microbial community, with its higher bacterial diversity and different non-yeast bacteria as compared to the microbiota involved in the fermentation of other styles of baijiu, resulting in the diversity of metabolites. It is concluded that the balance of interactions between microbial communities in jiuqu is of great significance to improve microbial quality, ensure its stability and ensure the quality of light-flavored baijiu.

#### Rice-Flavored Baijiu

Rice-flavored baijiu is a rare and one of the four basic flavor types of baijiu in China. The most representative is Guilin sanhua baijiu. Rice-flavored baijiu is a Xiaoqu baijiu made from rice. Its production consists of raw material pretreatment, inoculation, saccharification, fermentation, distillation, aging, and other processes. It tastes like honey, light and soft. The aromatic composition of this baijiu is mainly composed of phenyl ethanol, balanced with ethyl acetate and ethyl lactate. Among the four basic flavor types, rice-flavored baijiu contains the highest content of phenylethanol. The total alcohol content of rice-flavored baijiu was higher than that of ester compounds, and the ratio of total acid, total ester, and total alcohol was 1:1.2:1.5. In addition, lactic and acetic acids account for more than 90% of the total acids. Among the ester compounds, ethyl lactate has the highest content, followed by ethyl acetate (Yin et al., 2020b).

Rice-flavored baijiu is made of Xiaoqu, which acts as a microbial starter. Various functional microorganisms provide hydrolytic enzymes, volatile substances, and other components for baijiu brewing, which thus form the unique flavor of the baijiu (Wu et al., 2019; Sakandar et al., 2020). In the process of brewing rice-flavored baijiu, bacteria and fungi are involved. The primary microorganisms originate from the environment and the raw rice materials, which mainly included yeast and *Rhizopus* (Hu Y.L. et al., 2021). Compared with Maotai-flavored and Luzhou-flavored baijiu, relatively few types of microorganisms participate in the brewing process for rice-flavored baijiu, but a few microbial species dominate this brewing process, and they contribute flavor substances to rice-flavored baijiu.

#### Miscellaneous-Flavored Baijiu

Combined-flavored baijiu, also known as compound-flavored baijiu and mixed baijiu, refers to baijiu with more than two main aromas, and one baijiu with multiple aromas is its style feature. The production process of miscellaneous-flavored baijiu is unique, with a brewing process that includes starter fermentation, stacking fermentation, cellar alcohol fermentation, and grain distillation (Li et al., 2016). The most representative product of miscellaneous-flavored baijiu is Baiyunbian baijiu, which is the most popular mixed-flavored baijiu in China. Its sensory characteristics are between the sauce flavor and strong-flavored baijiu. The representative aromatic compounds are heptanoic acid, ethyl heptanoate, isoamyl acetate, octane, isobutyric acid, and butyric acid.

Some studies have continuously tested fermented baiyunbian baijiu, and the results show that the bacterial community is dominated by *Bacillus*, and *Lactobacillus*; the most common fungi are *Paecilomyces* spp., yeast, and *Zygomycetes cerevisiae*. A large number of fungi were from the genera *Thermomyces*, *Aspergillus*, *Monascus*, and the subgenus *Isachenki*, as well as some prokaryotes belonging to the genera *Acetobacter*, *Lactobacillus*, and *Thermoactinomycetes*. Analysis by GC-MS showed that the distiller’s grains contained high concentrations of ethyl lactate, 2,3-butanediol, and ethyl caproate, which were mainly produced by the co-fermentation of *Lactobacillus* and yeast (Liu and Miao, 2020). Dong et al. (2022) found that the dominant bacteria (*Weissella* and *Leuconostoc*) were replaced by *Acetobacter* and *Gluconobacter*, and the dominant fungus (*Rhizopus*) was partially replaced by *Thermomyces* in the saccharification stage of Baijiu brewing. While, in the fermentation stage, the dominant bacteria were replaced by lactic acid bacteria, and the dominant fungus was completely replaced by *Thermoascus* and other three yeasts, including *Saccharomyces, Kazachstania*, and *Apiotrichum*. The formation of flavor substances (acetic acid, lactic acid, hexanoic acid, ethyl lactate, and ethyl lactate) was found to be closely related to *Kazachstania* and *Apiotrichum*.

#### Feng-Flavored Baijiu

In the production of feng-flavored baijiu, sorghum is used as the raw material, and medium-temperature Daqu or Fuqu and yeast made of barley and peas are used as the starter. Continuous glutinous rice can also be used, and fermentation occurs in a cellar that is not more than 1-year-old. The most representative product of feng-flavored baijiu is Xifeng baijiu, which is the originator and typical representative of this type of baijiu that is widely known in China. The aroma of honey is a typical sensory characteristic of feng-flavored baijiu, which originates from a unique production process, and its formation mechanisms are still unclear. The main aromatic components of feng-flavored baijiu are ethyl acetate, ethyl hexanoate, and isoamyl alcohol, and its ethyl acetate and ethyl hexanoate are in balance. Known throughout the world as the ‘Three Wonders’ and ‘Phoenix in baijiu,’ it has a wide range of representations and a profound mass base in the country. It has been reported that the aroma of pineapple in feng-flavored baijiu is closely related to the amount of four esters, five alcohols, and two acids. Appropriate proportions of ethyl lactate, ethyl hexanoate, and ethyl acetate are essential for the formation of typical feng-flavored baijiu flavors. Crucially, if the amount of ethyl lactate is too high, it will weaken the aroma of the baijiu and increase its astringency (Jia et al., 2022b).

#### Medicine-Flavored Baijiu

To produce medicine-flavored baijiu, it is necessary to adopt the method of making incense sticks, making fermented grains from Xiaoqu and Daqu fragrant grains, and adopting a double glutinous rice method or re-steaming method (that is, a stringing incense method) or the double-steaming method. This baijiu has a medicinal-like aroma, moderate sweetness and sourness, and a lasting aftertaste. Dongjiu is a typical representative of medicinal baijiu. Additionally, 95 types of Chinese herbal medicines are added to Xiaoqu preparations, and 40 types of Chinese herbal medicines are added to Daqu preparations.

Previous studies on winter baijiu mainly focused on the production process, microorganisms, organic acids, and the separation and identification of a small number of terpenes. In previous studies, it was shown that the characteristic aromatic components of medicinal-flavored baijiu can be summarized as having high amounts of total acid, alcohol, and ethyl butyrate, and a low amount of ethyl lactate. In addition, the total amount of acid and alcohol in this type of baijiu is higher than that of ester baijiu, which contains different aromatic components as compared to other types of baijiu. However, thus far, the characteristic aromatic components contained in medicinal-flavored dongjiu baijiu are not clear.

#### Te-Flavored Baijiu

This special-flavored baijiu is made of whole grain rice as the main raw material, medium- and high-temperature Daqu as the saccharification starter, and it is fermented, distilled, aged, and blended by the traditional solid-state method. Te-flavored baijiu has the characteristics of multi-type, multi-layered fragrance, full-bodied, harmonious, sweet, and mellow in the entrance, refreshing aftertaste, and no discordant or miscellaneous flavors. The most representative Te-flavored baijiu is Si’te baijiu from Jiangxi, China. Si’te baijiu contains a high concentration of ethyl acetate and ethyl caproate as the main aromatic compounds, which are balanced with heptanoate. This type of baijiu has a harmonious and rich flavor and light taste. The concentrations of ethyl propionate, ethyl valerate, ethyl heptanoate, and ethyl pelargonate in Si’te baijiu are higher than that in any other type of baijiu. The aromatic components of te-flavored baijiu have the following characteristics: rich in odd-carbon fatty acid ethyl ester, with the greatest quantity among all types of aromatic baijiu. The esters in special-flavored baijiu are mainly ethyl propionate, and also ethyl valerate, ethyl heptanoate, and ethyl pelargonate; volatile compounds are ethyl acetate and ethyl hexanoate, in equilibrium with heptyl ester (Fu et al., 2021).

#### Sesame-Flavored Baijiu

Sesame-flavored baijiu is an important baijiu style, and its representative is Jingzhi baijiu. Its production process includes steaming and slag continuation, use of a mud-bottomed brick cellar, large bran combination, and multi-microorganisms’ co-fermentation. The fermentation technology of sesame-flavored baijiu is characterized by high nitrogen ingredients, high-temperature accumulation, high-temperature fermentation, and long-term storage. Modern biotechnology and traditional Daqu are combined with the use of bacteria, yeast, and mold for the fermentation production of sesame-flavored baijiu, which is an innovative improvement to the production of traditional baijiu. Sesame-flavored baijiu exhibits the ‘purity’ and ‘elegance’ of light-flavored baijiu, the ‘softness’ and ‘fullness’ of strong-flavored baijiu, and the ‘refinement’ and ‘nuances’ of Maotai-flavored baijiu. The comprehensive sensory evaluation indicates that the typical aroma of sesame-flavored baijiu is that of roasted sesame seeds (Yuan, 2009; Su et al., 2015; Sun et al., 2018a). Although there has been a great deal of research on sesame-flavored baijiu, their characteristic flavor compounds and their associated microbial metabolic mechanisms remain unclear. However, in recent years, research on sesame-flavored baijiu has been increasing, and it has been reported that some sulfur compounds in roasted sesame seeds have been identified as the source of the sesame-like aroma. Eleven sulfur-containing compounds have been identified in sesame-flavored baijiu, including dimethyl disulfide, trisulfide dimethyl ester, 3- (methyl sulfur) propionaldehyde, furfural mercaptan, and furfural disulfide. Therefore, sulfur compounds play a key role in sesame-flavored baijiu because they confer its roasted sesame-like aroma (Tamura et al., 2010, 2011; Yang et al., 2014).

#### Chi-Flavored Baijiu

Chi-flavored baijiu originates from the Pearl River Delta region, and it is named for its prominent soy flavor. Pure rice is used as the raw material, and large baijiu cakes act as saccharification starters in the fermentation process. The most representative soy flavor is Yubingshao baijiu, which is also called Xiaoqu baijiu. Its flavor is similar to that of fermented soybeans, with a very clean aftertaste. The main aromatic components of this baijiu are phenyl ethyl alcohol and ethyl ester. Testing of the microorganisms in the baijiu balls, baijiu cakes, and fermentation broth of soy-flavored baijiu revealed that there were 29 species of bacteria, of which *Lactobacillus* and *Halomonas* were the dominant flora. Additionally, there were 21 species of fungi, of which *Saccharomyces* and *Mucor* were the dominant bacteria.

#### Laobaigan-Flavored Baijiu

To produce Laobaigan-flavored baijiu, medium-temperature Daqu composed of pure wheat is necessary as the saccharification starter, with selected sorghum as the main material. The fermentation process involves ground tank fermentation, mixed steaming, segmented baijiu picking, graded storage, and careful blending. It has the characteristics of a short fermentation period, high production rate, and short storage period. Continued (Micha) mixed burning increases the utilization rate of starch, increases the yield, and steams the grains and baijiu at the same time, which increases its grain aroma. Its most representative product is Hengshui Laobaigan, which has a soft, mellow, and rich taste. The representative ester compounds in Laobaigan-flavored baijiu are ethyl acetate, ethyl lactate, and a small amount of ethyl butyrate, palmitate, and linoleic acid. The concentration of hexyl acetate is higher than that in light-flavored baijiu and Feng-flavored baijiu. The amount of fusel oil, especially isoamyl alcohol (7.17 mg/100 mL) in laobaigan-flavored baijiu is higher than that in light-flavored baijiu (flavored baijiu, 28.89 mg/mL), which enhances its sweetness and soft taste (Zhu et al., 2015; Wang et al., 2021).

#### Fuyu-Flavored Baijiu

The brewing process for fragrant-flavored baijiu is the inheritance and development of traditional baijiu production technology in China and has important practical significance for promoting the progress of traditional baijiu technology in China. Shen Yifang, the master of Chinese Baijiu, once highly evaluated the craftsmanship of jiugui baijiu and rich-flavored baijiu: ‘Although there are many flavor types, in the final analysis, the main ones are strong, clear, and sauce-like. Jiugui baijiu combines these three, and the combination creates a rich fragrance, which is an innovation.’ The representative product of fuyu-flavored baijiu is Jiugui baijiu, which has the sensory characteristics of clear fragrance, strong fragrance, and beige baijiu. The main aromatic compound is ethyl caproate, which contains equal amounts of ethyl lactate and ethyl acetate. By high-throughput sequencing, 11 bacterial genera were isolated, including *Bacillus*, *Lactobacillus*, *Leuconostoc*, *Weisseria*, *Lactococcus*, and *Acetobacter*; 8 yeast genera were isolated, including *Candida*, *Pichia*, and *Saccharomyces*, with *Lactobacillus*, *Welmanella*, and *Bacillus* in the bacterial community and *Rhizopus*, *Candida*, and *Pichia* in the fungal community, with *Aspergillus* predominating (Kang et al., 2021).

## Formation of Flavor Substances in Baijiu

The brewing process for baijiu is conducted in an open environment, with grain as the fermentation substrate and jiuqu as the saccharification starter. In the process of jiuqu production, the factors affecting the diversity and richness of the jiuqu microbial community include raw materials, production temperature, water, and so on. These bacteria, molds, and yeasts from their natural habitat all play a key role in jiuqu (Xiu et al., 2012). There were significant differences in microbial diversity among the three starter cultures, and the microbial community structure in starter cultures was the key factor determining the flavor diversity. It is reported that the bacterial species in Daqu and Fuqu are mainly thermophilic or heat-resistant. Fuqu and Xiaoqu both contain mold and *Rhizopus oryzae*. *Saccharomyces cerevisiae* and *Saccharomycopsis fibuligera* are also used in Fuqu and Xiaoqu, respectively. During the brewing process, the interaction of various microorganisms will produce the corresponding lipidosis, which plays a catalytic role and hastens the brewing process.

### Microbial Starter-Jiuqu

**FIGURE 2:**
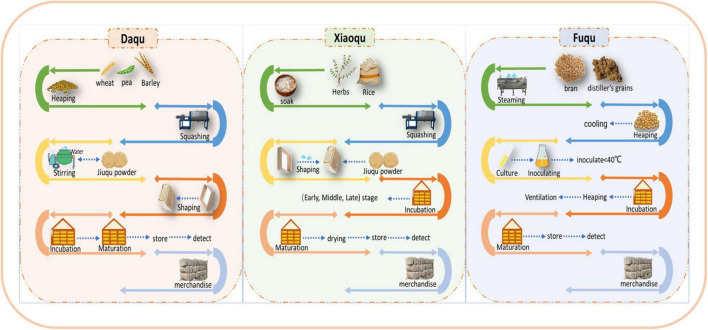
The production process of the three kinds of jiuqu.

Chinese Baijiu is produced through a solid-state fermentation process using a saccharified grain starter. Jiuqu provides raw materials, microorganisms, enzymes, and aromatic precursors for the brewing process. The production of Chinese Baijiu is the interaction of jiuqu and specific microflora in the environment, plus different materials and processing processes, creating different types of baijiu, which are also inseparable from the formation of flavor substances (Jin et al., 2017). According to the different production processes, jiuqu can be divided into three categories: Daqu, Xiaoqu, and Fuqu (Zheng and Han, 2016). The production process of the three kinds of jiuqu is shown in Figure 2.

Daqu is a grain distiller’s jiuqu, made from wheat (the sauce-flavored type is pure wheat), barley, and/or peas, providing mainly a sauce-like roasted aroma and pleasantly fruity, floral aromas. Daqu is further categorized into high-temperature Daqu (Maotai-flavored baijiu), medium-temperature Daqu (strong-flavored baijiu), and low-temperature Daqu (light-flavored baijiu) (Guan et al., 2020). High-temperature Daqu is divided into three kinds according to different positions in the workshop (white-Qu, yellow-Qu, and black-Qu). White-Qu had the highest liquefaction and saccharification enzyme activity, while black-Qu had the highest neutral protease and cellulase activity among the three kinds of high-temperature Daqu. The total volatile content of yellow-Qu and black-Qu is about twice that of white-Qu. Studies have shown that there are significant differences in microbial communities and metabolites among the three Daqu (Shi et al., 2022). The production process of Daqu is relatively complicated, whereby 70% of Chinese Baijiu are brewed with Daqu starter, including the most well-known baijiu in China, such as Maotai, Luzhou laojiao, and fenjiu, which are Maotai- flavored baijiu, Luzhou- flavored baijiu, and light-flavored baijiu, respectively (Wang et al., 2020b). Many intrinsic and extrinsic factors in Daqu production affect the microbial community richness and structure, including feedstock species, moisture content, temperature control, geographic location, climate, and moisture (Song et al., 2017). Daqu mainly consists of three functional microbial communities, namely Filamentous fungi (*Rhizopus, Rhizomucor, Aspergillus*, and other genera), Yeasts (*Saccharomyces, Candida, Hansenula*, and other genera), Bacteria (acetic acid bacteria, lactic acid bacteria, and *Bacillus* spp.) (Zheng et al., 2011). They provide different enzymes and flavor precursors for baijiu production. A previous study found that fungi, lactic acid bacteria, and *Bacillus* are the main flora in Daqu production. As the relative abundance of lactic acid bacteria increases, the acidity of Daqu also increases. In addition, Bacillus and thermophilic fungi become the dominant flora.

Xiaoqu is suitable for the climatic conditions of southern China owing to its small size and less heat generation. The production process of Xiaoqu is relatively simple. It is made from rice, sorghum, or barley and several traditional Chinese medicines. In the process of brewing baijiu, saccharification and fermentation occur at the same time in Xiaoqu, and the brewed baijiu is called Xiaoqu, which is different from Daqu. There are only a few types of microorganisms in Xiaoqu, including *Rhizopus*, *Mucor*, *Lactobacillus*, and yeast. These microorganisms are the executors of fermentation because only a small amount of starter and a shorter period are required to produce this type of baijiu. During the fermentation cycle, the baijiu contains less alcohol. Owing to the simple production process of Xiaoqu, the diversity of microorganisms is reduced, mainly *Saccharomyces cerevisiae* and *Saccharomycopsis fibuligera* (Zhou, 2011; Jin et al., 2017).

In Fuqu, bran is used as the raw material, and it is inoculated with jiuqu enzymes (*Aspergillus flavus*, *Aspergillus niger*, and *Aspergillus albus*), which mainly plays the role of saccharification, and then is cultivated by artificial control of temperature and humidity (Dai et al., 2020). When brewing, Fuqu also needs to be mixed with yeast (heat-resistant yeast, lipogenic yeast) for alcoholic fermentation. Fuqu has been successfully used to produce Maotai-flavored (Dai et al., 2020), light-flavored (Zhang et al., 2013), and sesame-flavored Chinese Baijiu (Wang et al., 2017a). Sesame-flavored baijiu is a new flavor produced with Fuqu as the main leavening agent and with Daqu. There is a saying in the baijiu industry that ‘Jiuqu is the bone of baijiu,’ which means that the type and quantity of microorganisms in baijiu determine the structure of the flavor substances in it, which verifies the importance of microbes in the process of brewing baijiu. Table 2 lists the basic characteristics of each jiuqu.

**TABLE 2:** Basic characteristics of each jiuqu.

Jiuqu	Types	Baijiu	Raw material	Microorganism species	References
High-temperature Daqu	Maotai-flavored baijiu	Moutai, Langjiu	Mostly made from wheat, barley, and/or peas; Maotai-flavored Daqu is only made from wheat	Some fungi, lactic acid bacteria; *Bacillus* are the main flora in Daqu production. *Ascomycota* and *Basidiomycota* were the dominant phyla	Jin et al., 2017; Song et al., 2017; Guan et al., 2020; Wang et al., 2020b; Cai et al., 2021
	Te-flavored baijiu	Si’te-jiu			
Medium/high-temperature Daqu	Strong-flavored baijiu	Luzhou laojiao, Wuliangye			
	Feng-flavored baijiu	Xifengjiu			
Medium-temperature Daqu	Laobaigan-flavored baijiu	Laobaigan			
Xiaoqu	Rice-flavored baijiu	Sanhua jiu	Rice, sorghum, and barley are used as main raw materials, and several traditional Chinese medicines are added.	*Rhizopus*, *Mucor*, *Lactobacillus*, and some Yeast, e.g., *Saccharomyces cerevisiae*, and *Saccharomycopsis fibuligera*	Zhou, 2011; Xiong et al., 2014; Jin et al., 2017
	Chi-flavored baijiu	Yubingshao jiu			
Daqu Xiaoqu mixture	Medicine-flavored baijiu	Dong jiu			
	Fuyu-flavored baijiu	Jiugui jiu			
Daqu and Fuqu mixture	Sesame-flavored baijiu	Jingzhijiu	Fuqu: bran as raw material	*Aspergillus flavus*, *Aspergillus niger*, some thermotolerant yeast, and lipogenic yeast	Jin et al., 2017; Wang et al., 2017a; Dai et al., 2020
Daqu (high-temperature Daqu/medium-temperature Daqu)	Miscellaneous-flavored baijiu	Baiyunbian	−	*Salmonella, Penicillium, Aspergillus, Streptococcus, Saccharomyces cerevisiae, Ssaccharomycopsisibuligera, Pichia pastoris* and *Debaryomycesansenii*	Xiong et al., 2014; Dong et al., 2022; Shi et al., 2022
Daqu, Xiaoqu, and Fuqu mixture	Light-flavored baijiu	Fenjiu		*Bacillus, Lactobacillius*, *Acetobacter* and *Lactococcus*	Dong et al., 2020; Su et al., 2020; Hu Y.A. et al., 2021; Tang et al., 2022

### Lipase Produced by Microorganisms

Esterase is a general term for a lipolytic enzyme, and they are very important in the brewing process of baijiu and mainly plays a catalytic role. Esterase in baijiu production mainly refers to lipase, ester synthase, and phosphatase as an extracellular enzyme that can catalyze ester synthesis and ester decomposition, which is mainly produced by microorganisms in the fermentation system. Most of the esterase-producing microorganisms are molds, such as *Aspergillus*, *Rhizopus*, and *Mucor*. Esters are important aromatic substances in baijiu. A large number of studies have found that various strains such as *Aspergillus niger* and *Rhizopus* have a strong ability to catalyze the production of fatty acid ethyl ester (Xu et al., 2021a). It was also found that Daqu is a saccharification agent and starter for brewing different types of baijiu. Because there is an increase during the brewing process in a large number of microorganisms that produce a variety of enzymes under the long-term open operating environment, Daqu not only contains abundant brewing microorganisms, but also contains enzymes including esterase, and therefore, Daqu is a very important flavoring agent (Nicholson, 2008). According to research data, the correlation between various enzymes and the production of total esters in baijiu is very small, while the correlation between various enzymes and the production of ethyl acetate, ethyl butyrate, ethyl hexanoate, and ethyl lactate is very different because the effect of the enzyme on total ester production reflects the combined effect of the enzyme on the production of various esters.

During the fermentation process of Chinese Baijiu, enzymes produced by microorganisms in the fermented grains decompose high molecular weight substances such as proteins, carbohydrates, and fats into low molecular weight substances such as amino acids, oligosaccharides, and fatty acids. Microorganisms utilize small molecules as nutrients and provide abundant precursors for flavor substances. The activity of enzymes not only affects the transformation of various substances in fermented grains but also directly affects the type and quantity of flavor substances, which has a profound impact on baijiu. For example, during fermentation, esterases directly catalyze the esterification of acetic and propionic acids with ethanol to synthesize ethyl acetate and ethyl propionate (Yu et al., 2012). According to Guo et al. (2018), increasing the lipase activity in fermented grains can increase the total ester content in baijiu, and adding cellulase to fermented grains can increase the ester content, especially ethyl acetate. The main application of esterase in baijiu production is to alter the production of various synthesized lipids, which in turn has an important impact on the formation of baijiu quality and its aroma. Therefore, the catalytic conditions under which different esterases perform have great guiding significance for their application in the brewing of baijiu.

### Microbiological Compositions

An enzyme is a biological catalyst that is mainly derived from microorganisms in the process of brewing baijiu. Most baijiu is fermented by a variety of microorganisms that can produce carboxylate hydrolase. Enzymes originate from ethanol and sour substrates in the baijiu production process (Zheng et al., 2011, 2014). The study of enzymatic synthesis of lipids began as early as the 1990s when a Dutch company launched a series of lipid products. United States federal regulations and some non-Chinese literature also recognize that biosynthetic lipids can be used as natural flavors. In a simple enzymatic reaction, enzymes catalyze the synthesis of ethyl ester from anhydrous ethanol and acid in different organic media. Compared with the ester chemical synthesis method, this method produces less environmental pollution with simpler post-processing of the product. Enzymatic synthesis has the advantages of mild reaction conditions, high conversion rate, reusable enzymes, mild product flavor, and natural aroma. It has important research significance and application value.

The reaction environment of enzymatic synthesis can be divided into lipidation reactions in a reverse micelle system and a single environment. A reverse micelle is a microemulsion, which is a surfactant dispersed in a continuous organic phase. The film formed by the surfactant can separate oily and aqueous substances, simulating the natural environment of enzymes, and thus assisting in maintaining the activity of enzymes and stability. The researchers discovered a novel catalyst by combining genome sequencing, and transcriptome sequencing techniques. This enzyme may promote the synthesis of ethyl valerate, ethyl caproate, ethyl capryate, or ethyl decanoate (Xu et al., 2021b). The discovery of new biocatalysts is of great significance in increasing enzyme sources for the synthesis of short chain fatty acid esters in the aqueous phase. In recent years, there has been great interest in the catalysis of biological enzymes in the organic phase. In organic solvents containing trace amounts of water, an enzyme will not lose its activity, and it also increases the protein’s catalysis due to the strengthening of intramolecular hydrogen bonds. Rigidity significantly increases the thermal stability of the enzyme and increases the solubility of non-polar substrates, which can reduce reaction byproducts and increase the reaction rate of synthesis.

### Microbial Synthesis

The Chinese Baijiu brewing system contains a complex microbial flora, consisting of a variety of bacteria, yeasts, and molds. Daqu is the source of the main microbial bacteria, molds, and yeasts, with the number of bacteria being the largest and relatively stable; the abundance of molds is relatively high, followed by yeasts (Guo and Jia, 2014). The essence of baijiu brewing is the process of microbial growth and metabolite accumulation, and therefore, the synergy between different microbial populations is closely related to the flavor and quality of baijiu (Yu et al., 2014).

The primary compounds in light-flavored baijiu that provide flavor are ethyl acetate and ethyl lactate. Lactic acid bacteria are the dominant microbial group in fragrant baijiu-fermented grains, and they are relatively stable in number during the fermentation process. They metabolize lactic acid and acetic acid, which are the precursors of ethyl acetate and ethyl lactate, to maintain an acidic brewing environment and provide other fermenting microorganisms that are precursors of nutrients and flavor compounds. During the process of brewing Xiaoqu baijiu, the starch in sorghum, which is an important substrate for microbial metabolism, is decomposed by microorganisms and converted into reducing sugars. *Saccharomyces cerevisiae*, which is an ester-producing yeast, and lactic acid bacteria utilize reducing sugars for proliferation and fermentation to produce metabolites, which result in the production of ethanol, ethyl acetate, and other important aromatic substances in jiuqu (Kerjean et al., 2000; Zha et al., 2018). Pang et al. (2020) found that *Lactobacillus* and *Pichia* were the main bacterial and fungal genera in the pretreatment process of the materials used for making light flavored baijiu. And with the dynamic change of microbiota in fermentation, the types and concentrations of alcohols, esters, acids, phenols, ketones, aldehydes, and other volatile flavor substances also varied. Environmental conditions and microorganisms have always been important factors driving the production of flavor substances in baijiu (Chen et al., 2021). A study found that the amount of esters and the acidity of light-flavored baijiu were significantly reduced when production was resumed after the summer break (Pang et al., 2018). Lack of acetyl resistant Lactobacillus may lead to delayed fermentation and lower the ester content. Environmental conditions (moisture, reducing sugar, and starch) and yeast are important factors in the brewing of light-flavored baijiu that affect the flavor substances in the fermented grains of Xiaoqu baijiu and promote changes in the composition and amount of flavor substances during different fermentation periods. During this process, with the change in the microbial community brewing structure, the flavor substances produced by its metabolism also changed.

Ethyl caproate is a strongly aromatic substance in Luzhou-flavored baijiu, and it is mainly produced by the high esterification of microorganisms such as aroma-producing yeast, bacteria, and mold in the mud pit in the later stage of fermentation. In the early stage of brewing, Luzhou-flavored baijiu mainly produces various acids and alcohols. In the middle and late stages, the acids and alcohols undergo biochemical reactions under the conditions of enzymes and appropriate temperatures to gradually generate various esters, which can form a unique style of baijiu (Liu et al., 2017). It has been reported that the quality of the Luzhou-flavored baijiu is closely related to the pit mud and the *Clostridium* spp. inhabiting the pit mud can produce caproic acid and ethyl caproate. At present, hexanoic acid and ethyl hexanoate have been identified as the key flavor substances in Luzhou-flavored baijiu (Guo and Jia, 2014; Li et al., 2014). Chen et al. constructed a recombinant strain EY15 by studying overexpression of EHT1 (encoding ethanol caproyltransferase) in which FAA1 (encoding acyl-CoA synthase) was deleted, followed by liquid fermentation of corn hydrolyzate and solid fermentation of sorghum. The yield of ethyl caproate was significantly increased to 2.23 and 2.83 mg/L, which were 2.97 times and 2.80 times that of the parental strain AY15, respectively. In addition, there was increased production of ethyl caprylate (52 and 43%) and ethyl caprate (61 and 40%) (Chen Y.F. et al., 2014).

During the saccharification process of brewing rice-flavored baijiu, the microorganisms and enzymes in Xiaoqu can degrade the starch in the rice into sugar (Devi and Radhika, 2018; Yin et al., 2020a). The main aromatic substances of rice-flavored baijiu are ethyl lactate and ethyl acetate, and the precursors of these two compounds are lactic acid and acetic acid. Relevant research analysis shows that in the brewing process of rice-flavored baijiu, bacteria and yeast genera have a great impact on flavor (Wang et al., 2017b; Gha et al., 2019; Xu et al., 2021b). Lactic acid bacteria (LAB) dominate the late fermentation stage and influence the sensory properties of baijiu by synthesizing flavor compounds that result from modulating the composition of other bacteria and yeasts. When there are acetic acid-tolerant lactobacilli participating in fermentation, the production of ethyl acetate, ethyl lactate, ethanol, acetic acid, and 2,4-di-tert-butyl-phenol can be significantly increased, and the lactic acid bacteria and the esters, acids, and flavor substances such as alcohol showed a strong positive correlation. However, in general, the microbial community diversity in rice-flavored baijiu is relatively higher than that of other types of baijiu during fermentation, because the different non-yeast composition leads to a diversity of metabolites.

Chinese Baijiu is rich in aromatic substances. Except for the characteristic aromatic substances in Luzhou-flavored, light-flavored, and rice-flavored baijiu, the characteristic flavor substances of other types of baijiu are still under consideration. Researchers are searching for new methods to increase the quality of baijiu and benefit human health by studying certain flavor substances. 2,3,5,6-Ligustrazine (TMP) is an important component in baijiu, and it has the effect of promoting cardiovascular and cerebrovascular health. During the process of brewing baijiu, the microorganisms in the jiuqu will produce acetone and then synthesize tetramethylpyrazine (TTMP), but the yield is low. Using the 2,3-butanediol dehydrogenase (BDH)-encoding gene BDH1 and another BDH2 gene deleted or overexpressed, Cui et al. (2020) showed that by disrupting BDH1 and overexpressing BDH2, acetoin synthesis in strain α5-D1B2 was significantly enhanced, resulting in a 2.6-fold increase in TTMP production.

## Action Mechanisms of Microorganisms in Baijiu Fermentation

### Saccharomycetes

Saccharomycetes is a single-celled fungus, classified as a facultative anaerobic microorganism, and it can ferment sugars to produce alcohol. Most of them survive in an environment with high sugar concentration and acidity, and they play the role of alcoholization and esterification in the process of brewing baijiu (Liu and Miao, 2020). Saccharomycetes has a variety of enzyme systems, which is also rich in protein, amino acids, fats, carbohydrates, and other nutrients. Generally, under anaerobic conditions, yeasts convert sugars into pyruvate through a series of enzymatic reactions through the sugar metabolism pathway, and finally into ethanol under the action of aldehyde dehydrogenase and alcohol dehydrogenase.

Studies have shown that yeast can produce aldehyde esters, β-phenethyl alcohol, and other higher alcohols during the fermentation process, and it also uses ethyl acetate as the main ester to form flavor substances (Li H.S. et al., 2021). There are two yeasts primarily used in baijiu brewing. One is *S. cerevisiae* (Wang et al., 2018b), which mainly has the functions of fermenting sugar, fermenting, and producing alcohol, and it has a relatively high alcohol-producing capacity. The other is lipogenic yeast, whose alcohol-producing capacity is smaller than that of *S. cerevisiae*. However, during the fermentation of baijiu, lipogenic yeast can convert some metabolites into aldehydes, esters, higher alcohols, and other compounds. Song et al. (2017) found that yeast is the core fungal microorganism in the fermentation process that produces Maotai-flavored baijiu. The origin of yeast can be traced back to the production stage of Daqu. During the process of fermentation, yeast and other microorganisms can promote and inhibit each other. Yeast not only directly determines the fermentation rate, but also converts nutrients into a variety of volatile flavor compounds. Therefore, yeast is one of the key factors affecting the flavor type and product quality of Chinese Baijiu. However, it is unclear whether other microbes possess similar interaction mechanisms. Therefore, while studying the diversity of microorganisms, the types and mechanisms of interactions between microorganisms are important because they can increase the quality of baijiu.

### Bacteria

Bacteria are prokaryotic organisms with a simple structure that undergo multiplication by binary division. Bacteria mostly survive in environments with suitable temperatures, high humidity, and saturated organic matter (Hu et al., 2015). In the process of brewing baijiu, bacteria are mainly used to produce flavor components and precursors of flavor components, which are important sources of the unique flavor of Chinese Baijiu (Tao et al., 2017). Bacteria in baijiu brewing are acid-producing microorganisms, and their metabolites can be used as precursors for lipid substances, and can also promote the growth and reproduction of other brewing microorganisms.

Aerobic and anaerobic bacteria, e.g., *Lactobacillus*, *Bacillus*, and *Clostridium*, participate in the process of brewing baijiu. The fermentation products of *Bacillus* mainly include 4 compounds, pyrazine compounds, volatile acids, aromatic compounds, and phenolic compounds, which are very important for Chinese Baijiu and significantly contribute to its aroma (Zheng et al., 2013). Some studies have found that the introduction of bacteria into Daqu and pit mud will affect the relative abundance of microorganisms, and thus, synthetic flavor compounds have been used to affect the sensory properties of different baijiu. Wang et al. (2017a) added the strain *Bacillus licheniformis* to the fermentation mix, and the microbial community subsequently changed after inoculation. It was noted that the number of *Bacilli*, *Corynebacterium*, and *Aspergillus* increased, and the number of *Pichia*, *Saccharomyces* and other genera decreased, which in turn altered the metabolic activity during the fermentation process. In the process of baijiu brewing, in addition to providing corresponding acid substrates, bacterial metabolism can also ferment glucose into ethanol through the Entner-Doudoroff (ED) pathway, but the efficiency is relatively lower than that of yeast. Bacteria can also metabolize components from complex and diverse enzyme systems, such as proteases, amylases, and cellulases. Bacteria decompose raw and auxiliary materials such as potatoes, rice, and sorghum during the baijiu-making process, and these are then formed into various amino acids, sugars, and other substances that continue to ferment and undergo biochemical changes to produce a unique flavor. In addition, Bacillus also promotes a variety of free amino acids to participate in the Maillard reaction, which endows baijiu with a unique flavor (Wang et al., 2018a).

### Mold

Mold was first recognized and utilized and is closely related to human life and material production. Mold is widespread, and its mycelium is composed of branched or unbranched hyphae and is relatively lush. Mold spores possess strong stress resistance and produce colonies that are loose and dry, in the form of spider webs, or fluffy, felt-like material (Hu et al., 2015). According to reports (Wang et al., 2008; Pang et al., 2018; Wang and Xu, 2019; Xnp et al., 2021), many fungal species such as *Aspergillus*, *Rhizopus*, *Mucor*, and *Penicillium* participate in the fermentation of cheese, baijiu, soy sauce, and other foods. *Rhizopus* produces glucoamylase and protease and some important flavor substances such as lactic acid, other organic acids, and aromatic compounds.

Studies have shown that starch in raw materials for brewing cannot be directly utilized by most yeasts and bacteria, and it must be hydrolyzed into fermentable sugars by α-amylasecuicui and saccharification enzymes produced by filamentous fungi during the process of saccharification and fermentation. The role of filamentous fungi is to secrete various enzymes to hydrolyze starch and protein to increase the digestibility and amount of probiotics in food and beverages (Wei et al., 2021). In the process of brewing baijiu, the brewing environment, various raw materials, and the mold in Daqu may all participate in the fermentation process, but the mold in Daqu plays the primary role. The saccharification power of mold can decompose the starch contained in the raw materials of brewing into reducing sugar that can be used by microorganisms. Mold possesses liquefaction power and esterification power, and it can also promote the decomposition and transformation of starch, protein, and other substances in the fermentation broth. This increases the concentration of sugars and amino acids throughout the fermentation process. Mold supplies nutrients for the metabolism of microorganisms in the fermentation system, and it also provides a unique flavor for the baijiu.

## Using Microbial Technology to Improve Baijiu Flavor

The isolation, research, reproduction, and reuse of microorganisms in baijiu brewing can greatly improve the performance of jiuqu, improve the quality and yield of baijiu, shorten the production cycle, and reduce costs. In addition, microorganisms can also be applied in some related fields.

### Jiuqu Strengthening

Based on microbial diversity research, isolated functional strains have been used to improve the quality of Daqu. *Lactobacillus*, *Aspergillus*, *Candida*, *Xanthomyces*, *Deba* spp., *Oosporium*, *Penicillium*, *Pichia*, *Saccharomyces*, and other strains have been added to improve the quality of Daqu during its production. Liu and Sun (2018) used red yeast rice or a mixture of fungi, yeast, and bacteria to fortify Daqu, and the results showed that the use of fortified Daqu increased the quality and yield of the produced baijiu, while decreasing the necessary amount of Daqu, which resulted in lower costs. Daqu fortification has the advantages of convenient operation, easy preparation, time-saving, and satisfactory batch-to-batch stability. In addition, the flavor and texture of the baijiu brewed with this new type of Daqu are comparable to those brewed with the traditional Daqu. These studies reveal the importance of microbial community investigation and the applicability of microbial resources in baijiu brewing to improve process control.

### Controlled Fermentation

Traditional solid-state fermentation tends to lead to batch-to-batch instability of the product, which is a bottleneck that needs to be addressed. Given the importance of microorganisms in the fermentation process, there has been great interest in the use of functional microorganisms to manually regulate the fermentation process. Wang et al. (2020e) simulated solid fermentation by adding *Wickerhamomyces anomalus* and showed that *W. anomalus* altered ethyl acetate content and caused changes in levels of other flavor substances. The results showed that the flavor changes caused by the addition of *W. anomalus* were due to the change in fermentation microbial community structure. Researchers have improved the flavor of baijiu using exogenous microorganisms to modulate the composition of the microbial community. The effect of exogenous microorganisms alters the growth and flavor metabolism of endogenous microorganisms, which suggests the possibility of using exogenous microorganisms to modulate the microbiota of the fermentation process. Further improvement and refinement will assist in maintaining product stability between batches and improve the quality of Chinese Baijiu (Fan and Qian, 2006).

### Cellar Mud Maintenance

The pit mud produced by Chinese Baijiu is currently aging and degraded, and this is caused by the consumption of nutrients by microorganisms and the accumulation of secondary metabolites, the inhibition of the growth of functional microorganisms, and the degradation of bacterial strains. All of these will lead to the degradation of pit mud and reduce the quality of baijiu. Laojiao assists in producing high-quality baijiu, and therefore, the maintenance of Laojiao mud is very important. There have been attempts to grow microorganisms such as *Clostridium* and add them to the pit mud. The results showed that the amount of ethyl caproate was increased, and the quality of Luzhou-flavored baijiu was improved (He X. et al., 2020).

Researchers have isolated many functional strains of microorganisms from pit mud, lees, and Daqu. In addition to the above-mentioned applications, strains such as *Monascus* and *Rhizopus* are also used in other fields. The red pigment produced by *Monascus* can be used as a food additive, and this species can also produce lovastatin, which is an effective drug for the treatment of cardiovascular and cerebrovascular diseases (Sun et al., 2018b). *Rhizopus* can synthesize oligosaccharides with various physiological functions, which can be used to produce healthy food. Other species produce a range of enzymes such as lipases, proteases, and amylases that can be used in related fields (Ma and Zhang, 2014). Through continuous research, additional strains with new characteristics will be discovered, and they will promote the study of baijiu-brewing mechanisms and also provide valuable resources for applications in many fields.

## Method for the Detection of Flavor Substances in Baijiu

The trace components in Chinese Baijiu only account for 1 to 2% of the total but have an important impact on baijiu aroma and taste. After years of research, it has been proven that the flavor composition of Chinese Baijiu is extremely complex, with many types and a wide range of content. Flavor component analysis of baijiu is performed mainly to detect trace components. Nearly 2,400 flavor compounds have been detected in baijiu thus far, including alcohols, esters, aldehydes, ketones, acids, acetals, aromatic compounds, lactones, furans, terpenes, hydrocarbons, nitrogen-containing compounds, and sulfur-containing compounds. This includes 216 types of alcohols, 431 types of esters, 95 types of aldehydes, 126 types of ketones, 109 types of acids, 54 types of acetals, 167 types of aromatic compounds, 19 types of lactones, and 76 types of furans and terpenes. Additionally, there are 68 alkenes, 81 hydrocarbons, 115 nitrogen compounds, 55 sulfur compounds, and 125 others (Sun et al., 2015). A variety of substances has been detected in baijiu, but the contribution and influencing mechanism of most flavor components are still unclear. At present, researchers can use conventional detection techniques, chromatographic techniques, spectroscopic techniques, and other analysis and detection techniques. Chromatography techniques are relatively common and accurate research methods, and a system has been built with these techniques for the detection and analysis of Chinese Baijiu (Jia et al., 2020a; Wu et al., 2020b). The chromatographic methods used to detect some flavor substances in Chinese Baijiu are summarized as follows, in Table 3.

**TABLE 3:** Chromatographic methods used to detect Chinese Baijiu flavor substances.

No.	Instrument	Type of baijiu	Flavor substances	Number of flavors	References
1	GC	Maotai-flavored baijiu, Strong-flavored baijiu, Light-flavored baijiu, Miscellaneous-flavored baijiu, Medicine-flavored baijiu	Organic acids, alcohols, esters, ketones, and aldehydes	62	He X. et al., 2020
2	GC-MS	Sesame-flavored baijiu, Light-flavored baijiu	Sulfur compounds: dimethyl disulfide, dimethyl trisulfide; propyl lactate; acetic acid; butyric acid; valeric acid, caproic acid	125	Ma and Zhang, 2014; Sun et al., 2017, 2018b
3	HPLC HPLC-Q-TOF-MS	Sesame-flavored baijiu	Esters and alcohols	1	Huang et al., 2019
4	SBSE and GC-MS	Maotai-flavored baijiu	Esters, alcohols, aldehydes, and ketones	76	Fan et al., 2011
5	GC-O	Strong-flavored baijiu	Ethyl esters, alcohols, aldehydes, acetals, alkyl pyrazines, furan derivatives, lactones, sulfur-containing compounds	126	Fan and Qian, 2006
6	GC-O and GC-MS	Strong-flavored baijiu, Light-flavored baijiu	Aromatic compounds: ethyl acetate, ethyl acrylate, ethyl isovalerate, ethyl butyrate, isoamyl acetate, ethyl caprylate, ethyl valerate	60/59	Zhao et al., 2018; Wang et al., 2022b
7	GC ×GC mil TOF/MS	Laobaigan-flavored baijiu	volatile chemicals	414	Fan Q. et al., 2019
8	MASTER GC/TOF MS	Strong-flavored baijiu	Organic acids, alcohols, esters, ketones, aldehydes, acetals, lactones, and nitrogen- and sulfur-containing compounds	528/262/606	Zhu et al., 2007; He Y.X. et al., 2020; Wang et al., 2020c
9	^1^H (NMR)	Light-flavored baijiu, Strong-flavored baijiu, Maotai-flavored baijiu	Mannitol, trimethylamine, lactic acid, etc.	−	Wu et al., 2009
10	GC and ^1^ H NMR	Fuyue- flavored baijiu Light-flavored baijiu, Strong-flavored baijiu	acetaldehyde, butyrate, valeric acid, n-butanol, 2-butanol, hexanol, ethyl butyrate, ethyl valerate, ethyl heptanoate and ethyl caproate	10	Li Y. et al., 2021
11	GC-MS and MIR	Strong-flavored baijiu	Propionic acid, pentanoic acid, hexyl hexanoate, ethyl decanoate, etc.	−	Hu and Wang, 2021
12	GC-O and GC-MS	Maotai-flavored baijiu	Ethyl acetate, ethyl lactate, acetic acid	79	Niu et al., 2016
13	GC-O and GC-FPD	Light-flavored baijiu	ethyl acetate, diethyl succinate, phenylethyl alcohol, isoamyl alcohol, and n-propanol, A tetrapeptide (Asp-Arg-Ala-Arg)	80	Niu et al., 2017
14	HS-SPME and GCxGC-SCD	Laobaigan-flavored baijiu	Benzenemethanethiol, Dimethyl trisulfide, 2-methyl-3-Furanthiol and 2-furfurylthiol exhibited, etc.	65	Song et al., 2019
15	HS-SPME and GC × GC-TOFMS	Strong-flavored baijiu, Light-flavored baijiu, Maotai-flavored baijiu	Esters, alcohols, fatty acids, aldehydes, furans, pyrazines, sulfides, phenols, etc.	119/19	Tang et al., 2022
16	HS-SPME-GC-MS/O	Daqu	aromatic compounds:guaiacol, 4-ethyl-2-methoxy phenol, 2-ethyl-3,5-dimethylpyrazine, etc.	43	Wang et al., 2022a
17	UHPLC-Q-Orbitrap HRMS/UHPLC-Q-Orbitrap	Feng-flavored baijiu	non-volatile molecule, 15 organic acids, 8 esters, Ethyl carbamate	153/29	Jia et al., 2020b,^2021^, 2022a
18	HS-SPME-AEDA	Sesame-flavored baijiu	Ethyl hexanoate, 2-furfuryl mercaptan, dimethyl trisulfide, 3-methyl butyraldehyde, ethyl butyrate, ethyl 2-methyl butyrate, ethyl valerate, and 4-methyl valeric acid ethyl ester	63	Sha et al., 2016
19	UPLC-MS/MS	Maotai-flavored baijiu, Strong-flavored baijiu, Light-flavored baijiu, Sesame-flavored baijiu	Pyrazines aroma compounds: 2,3-dimethylpyrazine, 2,3-diethylpyrazine, 2,3-diethyl-5-methylpyrazine and 2-acetyl-3-methylpyrazine	16	Yan et al., 2020, 2021
20	(Vis/NIR)	Various flavored baijius	−	−	Li et al., 2014

With the continuous development of science and technology, some novel detection methods have gradually emerged. For example, using simple organic reactions such as the hydroxylamine color reaction, 2,4-dinitrophenylhydrazine (DNPH) nucleophilic addition reaction, and localized surface plasmon resonance (LSPR) exhibited by noble metal nanoparticles, an eight-channel array sensor was constructed for the direct and qualitative detection of compounds such as aldehydes, ketones, esters, and acids in baijiu (Zhao et al., 2018).

In addition, some unscrupulous merchants have been illegally adulterating baijiu, such as adding sildenafil. It is difficult to detect sildenafil using ordinary methods, and its presence in baijiu can result in severe health effects. Xiao and He (2019) used Opto Trace Raman 202 (OTR 202) as a surface-enhanced Raman spectroscopy (SERS) active colloid to detect sildenafil, and the results showed that the Raman enhancement factor (EF) of the OTR 202 colloid reached 1.84 × 10^7^. Thus, the limit of detection (LOD) for sildenafil in health baijiu and baijiu was found to be as low as 0.1 mg/L. In recent years, there has been increased quality monitoring of Chinese Baijiu, and its analysis and testing have been developed for more than 50 years. There was an initial stage of development that progressed to a stable and mature stage, and now, analysis and testing have entered a new and modern stage of development with remarkable results. The progress of analytical technology, especially the application of chromatographic analysis technology, has greatly promoted the use of technology in the baijiu industry and made outstanding contributions to the inheritance and development of the traditional Chinese Baijiu industry.

## Summary

Chinese Baijiu has been exported all over the world, and it is increasingly recognized and appreciated in the international market. The production process of baijiu, including the production of jiuqu, has been modernized. However, owing to the wide variety of baijiu, the production technology for the different types must be updated based on retaining the original flavor. Therefore, understanding the ecology and function of each type of baijiu-related microorganism is the primary condition for realizing the standardization of baijiu production, and research on microorganisms plays a positive role in promoting the development of China’s baijiu industry. In the process of brewing baijiu, almost all strains promote the production of flavors such as esters, alcohols, and acids, and especially the formation of esters. Therefore, the species and even the strains of microorganisms in the brewing process of baijiu determine the flavor substances of baijiu. The balance and interaction of various flavors in baijiu depend on the balance and interaction of the microbiota during fermentation. With the development of microbiology, molecular biology, and bioinformatics technology, it is possible to further study the microbial community, functional strains, and their properties to reveal the mechanism of baijiu brewing and to discover the relationship between microorganisms and baijiu quality and yield. On this basis, we can also enrich the resource pool of functional strains, develop application technologies for concurrent functional strains, improve jiuqu production and grain fermentation processes, and adjust the fermentation process to produce quality products that meet consumer requirements. This will satisfy the development of the baijiu industry and promote the standardization and modernization of Chinese Baijiu.

## Author Contributions

WT, LL, and XC contributed to literature retrieval and information collection. TZ, QW, JC, PX, and WT analyzed the data. WT, QL, and CS wrote and reviewed the manuscript. All authors contributed to the article and approved the submitted version.

## Conflict of Interest

XC, CS, and QL were employed by Luzhou Laojiao Co., Ltd. The remaining authors declare that the research was conducted in the absence of any commercial or financial relationships that could be construed as a potential conflict of interest.

## Publisher’s Note

All claims expressed in this article are solely those of the authors and do not necessarily represent those of their affiliated organizations, or those of the publisher, the editors and the reviewers. Any product that may be evaluated in this article, or claim that may be made by its manufacturer, is not guaranteed or endorsed by the publisher.
